# Chemical Assessment of Drinking Water Quality and Associated Human Health Risk of Heavy Metals in Gutai Mountains, Romania

**DOI:** 10.3390/toxics12030168

**Published:** 2024-02-22

**Authors:** Thomas Dippong, Maria-Alexandra Resz

**Affiliations:** 1 Faculty of Science, Technical University of Cluj-Napoca, 76 Victoriei Street, 430122 Baia Mare, Romania; 2 INCDO-INOE 2000, Subsidiary Research Institute for Analytical Instrumentation, 67 Donath Street, 400293 Cluj-Napoca, Romania

**Keywords:** water chemistry, nutrient load, risk assessment through water ingestion, heavy metals pollution indices

## Abstract

Chemical data compiled from field and laboratory studies were analysed on drinking water sources from a mountain area (Gutai Mountains) in Romania. Six physico-chemical indicators, nine anions, and twenty-one metals were determined and analysed. The results of this study showed that waters are generally rich in NH_4_^+^ and NO_2_^−^, exceeding the recommended limit of 0.5 mg NH_4_^+^/L, while some waters are rich in As, Cd, Mn and Pb, but with concentrations below the limits concerning the use of waters with drinking purposes. The applied heavy metal pollution indices (scores: 0.56–47.9) indicate that more than 50% of samples are characterized by medium pollution degrees. Based on the results obtained, it was determined that geological and human activities were influential in enriching the studied waters with the chemicals considered. Emphasizing this aspect related to pollution sources and the importance of a clean chemical status that must characterize waters used for drinking purposes, a human health risk assessment for heavy metals was implemented. The results indicated that even though the studied waters are rich in heavy metals, scores related to the risk assessment of heavy metals indicated a lack of non-carcinogenic risks for As, Mn, Cd and Cu. Nevertheless, this study and the results obtained are significant at national and international levels by offering a perspective on determining the potential pollution and associated human health risks at heavy metals in drinking water sources from a mountain area.

## 1. Introduction

Water is a finite and essential element for human, plant and animal life. Commonly, it persists in three primary natural sources: fresh surface lakes, rivers and groundwater [1]. These water sources serve as a water supply for several geographical areas worldwide. In some cases, groundwater sources are the most dependable water sources for irrigation, household and other economic activities [2,3]. It has been estimated that approximately 30% of the world’s population uses groundwater resources, as they have the lowest share of pollution for drinking and domestic purposes [4,5]. 

Given the emergence of population growth and its consequences, rapid urbanization, intense industrial and agricultural activities with all the emissions and waste, toxins and excess chemicals occur in the environment, altering the properties of the air, soil and water [3,6,7].

Exposure through water ingestion or dermal contact pathways to certain toxins, heavy metals and nitrogen compounds, for example, alters human health, with acute or toxic effects [8]. For instance, water rich in chlorine can alter the rate of cell division and DNA structure and even hinder the reproductive system, which can destroy the organism [8]. The toxicity of arsenic (ubiquitous heavy metalloid, one of the essential heavy metals that worryingly affects the environment and animals’ health) is seen to impact the population’s health through accidental ingestion of some powders or solutions through water [9,10,11,12,13]. All heavy metals are natural elements; Fe, for example, represents about 5% of the Earth’s crust, is found as a compound, ferrous bicarbonate and is declared a rich Earth resource. It is found in the composition of water as insoluble ferric iron and soluble ferrous. It is dissolved in water, associated with oxygen or a disinfectant, and oxidation occurs. Compared to As, the adverse effects on human health are incomparable, causing itchiness and dryness to the skin [7]. Cd is considered the most toxic element, even at a low concentration. The primary source of high amounts of heavy metals in natural water systems is represented by industrial processes, such as metal distribution plants, stainless steel welding, tannery services and diverse manufacturing processes [14,15,16,17]. Mn is the most abundant metal in its natural state in the environment. Selenium has attracted some interest because it is an essential nutrient for humans within a limited range of daily intake, but it will turn into a toxin in high amounts [18,19,20]. Besides industry processes, nuclear activities are sources of chemicals with serious adverse effects on human health, such as the elements Rb, U and Po with radioactive properties [21].

In order to understand the reliability of waters used as drinking water sources, even if they are situated at significant distances from pollution sources, in mountain areas, for instance, it is mandatory to determine the water chemistry and investigate the potential pollution level, which also implies an understanding of the potential human health risk associated with heavy metals or other toxins. In this frame, consistent pollution indices and human health evaluation methodologies convert quantity data into quality data. The most used heavy metal pollution index, the heavy metal pollution index (HPI), and the heavy metal pollution evaluation index (HEI) are nominated. They are based on the maximum allowable concentrations (MACs) of the chemical parameters significant to be evaluated in order to assess the quality of drinking water. Water analyses through this method are classified into one of the specific pollution levels related to the applied individual heavy metals [22,23,24].

Applying these techniques, conventions and fixed values (reference doses for each heavy metal of interest) are used to estimate the potential non-carcinogenic and carcinogenic risks of heavy metals through water ingestion. There are few studies at national and international levels on water collected from mountain areas and analysed to determine the pollution level and associated human health risks; most studies focus on urban, mining or industrial areas. This aspect emphasises the novelty of this paper.

The objectives of this work, characterized by a consistent degree of novelty, are to identify and assess drinking water sources in the Gutai Mountains. Six physico-chemical indicators (electrical conductivity, pH, oxidation-reduction potential, total dissolved solids, turbidity and dissolved oxygen), eight anions (CO_3_^2−^, HCO_3_^−^, SO_4_^2−^, Cl^−^, Br^−^, PO_4_^3−^, NO_3_^−^, NO_2_^−^), NH_4_^+^, and twenty-one metals (Al, As, Ba, Cd, Co, Cr, Cu, Fe, Li, Mn, Ni, Pb, Se, Sc, Sr, Rb, Zn, Ca, Mg, Na, K) were determined. The chemical loading of drinking water sources was analysed, followed by calculating the potential contamination degree and risk assessment of heavy metals. The effects and causes of the presence of chemical indicators in water were studied. Based on the obtained results, the population needs to have awareness regarding the quality of untreated drinking water sources used for consumption and their harmful effects on health. By introducing innovative perspectives and methodologies, this study adds to the existing literature, seeking to enhance the knowledge base in the field. This understanding, in turn, can provide valuable recommendations for the population, aiding in health risk prevention and promoting sustainable practices.

## 2. Materials and Methods

### 2.1. Study Area Location and Sampling

**Figure 1:**
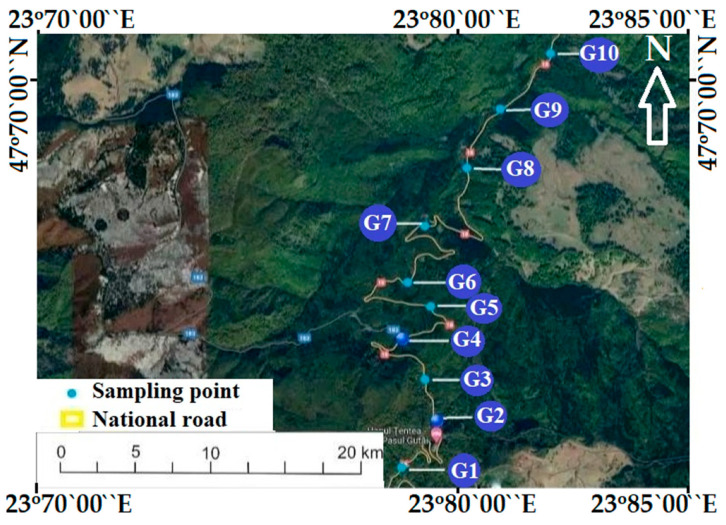
Sampling points (G1–G10).

Gutin Pass, Romania, is situated in the central–west part of Maramures, extending from the Gutin Peak (1443 m) to the Baii Mari depression. The mountainous area comprises over 60% of the scope of the studied area, formed by the southern peaks of the Igniș Mountains and the Lăpuș and Gutin Mountains, which are of volcanic origin. The depression area is made up of basins of complex origin. The Baia Mare depression is the most extensive, with an altitude of 200 m, and consists of piedmonts, glaciers, terraces and wide meadows. Ten sampling points (G1–G10) were established for groundwater (spring mountain water) used as a drinking water source in the Gutai Mountains (Figure 1). Twelve sampling campaigns (every month) were organized in 2022. In each campaign, 10 samples were collected, resulting in a total of 120 samples. Procedural standards [25] were followed to perform correct sampling. Samples were directly collected in polyethylene bottles after rinsing with the studied water. Portable equipment was used in-situ for the essential physico-chemical determinations. Duplicates of samples were acidified by adding nitric acid (65% supplied by Merck) until the pH reached 1.0 to 2.0. This prevents precipitation. Water samples were carefully transported and preserved for 24 h until the analysis.

### 2.2. Chemical Analysis

The chemistry of groundwater samples (G1–G10) was studied by measuring the following indicators: pH, EC—electrical conductivity, DO—dissolved oxygen, ORP—oxidation-reduction potential, T—turbidity and TDS—total dissolved solids, 9 anions (CO_3_^2−^, HCO_3_^−^, SO_4_^2−^, Cl^−^, Br^−^, F^−^, PO_4_^3−^, NO_3_^−^, NO_2_^−^), 9 cations (NH_4_^+^, Na^+^, K^+^, Li^+^, Ca^2+^, Mg^2+^, Sr^2+^, Ba^2+^ and Al^3+^) and 13 heavy metals (As, Cd, Co, Cr, Cu, Fe, Mn, Ni, Pb, Sc, Sr, Rb, Zn). 

The pH, OPR, EC and DO were measured in-situ with the help of portable equipment (Hach Lange SL1000, Loveland, CO, USA), considering the following standards [26,27,28]. SR EN ISO 7027/2001 [29] was followed to determine turbidity with the help of a turbidimeter (WTW, Troistedt, Germany). TDS was determined gravimetrically, while the content of Cl^−^, CO_3_^2−^ and HCO_3_^2−^ was determined titrimetric following standard procedures: American Public Health Association APHA (1999) [30,31]. PO_4_^3−^, NO_2_^−^, NH_4_^+^, F^−^, SO_4_^2−^ and NO_3_^−^ were analysed with the help of a Lambda 25 spectrophotometer (PerkinElmer NexlON 300S equipment) and portable equipment (Hach Lange HQ40d, Loveland, CO, USA). The trace elements and metals were measured through inductively coupled plasma–mass spectrometry (using Perkin Elmer NexlON 300S equipment, Norwalk, CT, USA). Quality assurance was conducted by measuring blank and replicates of samples and analysing standard solutions (reference and certified reference materials). The used reagents were supplied by Merck (Darmstadt, Germany) and are traceable to NIST SRM, and those used for trace elements determination were all of the analytical grade supplied. Monoelement and multielement ion solutions (1000 mg L^−1^) were used to validate and calibrate the ion chromatograph and inductively coupled plasma–mass spectrometer (n = 7-point calibration curves) equipment. For the validation of spectrophotometric analysis, 4-point calibration curves were implemented by preparing 8 standard solutions starting from an ammonium chloride reference material (assay > 99.8%). The determination coefficients obtained in calibrating all ion methods ranged between 0.9989 and 0.9999, indicating accurate methods. The detection limits for trace metals are below 0.01 µg/L, anions 0.05 mg/L, ammonia 0.03 mg NH_4_^+^/L and 10 mg/L for the TDS. The accuracy was determined by measuring certified reference materials, such as NIST 1643F for Trace Elements in water, the ammonium standard for ammonium determination, supplied by Merck, and SPS-NUTR WW1 for anion determinations, supplied by Spectrapure Standards (Oslo, Norway). The uncertainty of the methods was lower than 14%, and the recovery ranged between 90 and 120%.

### 2.3. Data Analysis

#### 2.3.1. Water Typology

TIS (Total Ionic Salinity), Gibbs and Piper plots were used to determine the water typology. Free versions of GWChart v 4 and XLStat Microsoft Excel software 2020 were applied for plotting. TIS studies water salinity given the ion concentrations (SO_4_^2−^, HCO_3_^−^ and Cl^−^) [32]. Based on the correlation between the lithological properties and water chemistry, the Gibbs plot was implemented, which analysis three processes and sources related to the water samples, such as evaporation–crystallization, rock interaction, and atmospheric precipitation [33,34]. Given the major ion content, the Piper diagram analyses the geochemical evolution of water. Samples are distributed in three different fields (cations, anions and the intersection files between cations and anions) showing the water typology [35,36].

#### 2.3.2. Heavy Metal Pollution Evaluation

Pollution and risk assessment of heavy metals was applied to determine the degree of heavy metal pollution and potential non-carcinogenic risks through the water ingestion exposure type. In general, mathematical tools are successfully used for assessing the level of heavy metal pollution. The most commonly used methods are the heavy metal pollution index (HPI) and the heavy metal evaluation index (HEI). These indices are worldwide, commonly and effectively used, and are calculated with the help of the following equations [22,24]:

(1) $$\mathrm{HPI}=\frac{\sum_{i=1}^{i=n}\left(q_{i}\times W_{i}\right)}{\sum_{i-1}^{i=n}W_{i}}$$

(2) $$q_{i}=\overset{n}{\underset{i=1}{\sum }}100\times \frac{C_{i}-I_{i}}{G_{i}-I_{i}}$$

(3) $$\mathrm{HEI}=\sum_{i=1}^{n}\frac{C_{i}}{G_{i}}$$

Here, W_i_ and q_i_ represent the determined pollutants’ unit weight and subindex. C_i_ and I_i_ are the studied heavy metals’ measured and ideal concentrations, while G_i_ is the threshold limit established for water used for drinking purposes [22,24]. In the present study, G_i_ was used according to the National Guideline Order 7 from 2023 and the International Guideline established by the WHO in 2017 [37]. Specific scores are obtained after calculating the pollution indices, defining the specific heavy metal pollution degree of water. There are three pollution degree categories: high (HEI ≥ 20 and HPI ≥ 30), medium (10 ≤ HEI < 20 and 15 ≤ HPI < 30) and low (HEI < 10 and HPI < 15) [22,24].

#### 2.3.3. Human Health Risk Evaluation

Non-carcinogenic risk assessment of specific heavy metals through ingestion was determined through defined indices, namely the hazard quotient (HQ), the chronic daily intake (CDI) and the hazard index (HI). According to Ogarekpe et al. [38], certain indices are calculated by applying the following equation:

(4) $$\mathrm{HQ}=\frac{\mathrm{CDI}}{\mathrm{RfD}}$$

(5) $$\mathrm{CDI}=\frac{\mathrm{HM}\times \mathrm{DI}\times \mathrm{EF}\times \mathrm{EP}}{\mathrm{BW}\times \mathrm{AT}}$$

(6) $$\mathrm{HI}=\sum {\mathrm{HQ}}_{\mathrm{HM}1}{\mathrm{HQ}}_{\mathrm{HM}\;2}\ldots {\mathrm{HQ}}_{\mathrm{HMn}}$$

Here, RfD represents the reference dose established for each heavy metal content by the US EPA and has the following values: 0.7 mg/kg/day for Cd, 3 × 10^−3^ mg/kg/day for As, 0.14 mg/kg/day for Mn and 0.04 mg/kg/day for Cu [39]. HM is the content of studied heavy metals (mg/L), and DI, EF, and EP are the average daily intake of water (2 L/day), annual exposure frequency (365 days/year) and exposure duration in the case of adults (70 years), while BW and AT are the average body weight of consumers (70 kg) and averaging time (EF × EP). HI is the hazard index, and HQ_HM1…HMn_ represents the hazard quotient calculated for each studied heavy metal [38]. If waters are characterized by HQ and HI scores exceeding the threshold of 1.0, potential non-carcinogenic risks to metals can occur through water ingestion [38].

## 3. Results and Discussions

### 3.1. Physico-Chemical Assessment of Studied Samples

Table 1 presents the physico-chemical composition of the studied water samples (G1–G10). The measured EC is generally low, below the MAC (2500 μS/cm), ranging between 68 and 663 µS/cm. G7 is characterized by the lowest EC, and G2 by the highest EC. High EC results in an ecological effect on the aquatic biota and is caused by water infiltration through soil and dissolving rocks. Generally, low EC is caused by precipitation, rainfall, and water–rock interaction [40]. EC reflects the salt content in water, so if consumed, water with high EC could lead to diverse diseases (cancer, diarrhoea, hepatitis or gastroenteritis) affecting the heart, kidneys and stomach [40]. The dissolved oxygen (DO) varied between 6.9 and 8.9 mg/L. G9 has the highest DO concentration, caused by aquatic microorganisms, which produce oxygen through photosynthesis and give the water a fresh taste. DO indicates the water stratification and the contamination degree [41]. The volume of DO in water varies depending on the water temperature, air pressure, microorganisms and oxidizable substances contained in the water. A low amount of DO leads to a lack of freshness, a bad taste and unfriendly conditions for microorganisms, which make the water not potable [6]. The high amount of DO increases the organic suspended matter rich in pathogens [6,41].

The pH ranged between 6.5 and 8.0, within the thresholds established for drinking water, indicating a weak acidic to weak basic character and indicating a low pollution level with organic and inorganic compounds [42]. Due to the water–rock interaction and rich concentrations of carbonates, the pH changes the taste of water and could cause skin and eye rashes [43]. The ORP varied between 179 and 234 mV, suggesting organic matter oxidation and decreasing the activity of microorganisms. High OPR is related to microorganisms, organic matter (dust, faeces, urine), chloramine and hypochlorite [24].

Turbidity ranged between 2.5 and 5.6 NTU. The presence of bacteria, plankton, iron and aluminium hydroxide, sludge and colloidal matter causes high turbidity. Water with high turbidity can cause an epidemiological hazard if consumed because the suspended material can support germs [44]. If waters with high turbidity are consumed, it could cause health issues, such as intestinal diseases [45,46].

Except for samples G3, G8 and G9, the rest of the samples are rich in NH_4_^+^, almost twice the MAC established for drinking water. The high amount is related to anthropogenic activities, for example, the intense use of fertilizers or faecal deposits. Waters increase in NH_4_^+^ amount if the aquatic organisms start to vanish. If consumed, waters with high amounts of NH_4_^+^ cause convulsion, hepatic encephalopathy, coma and even death. NH_4_^+^ can be reduced through energetic chlorination and filtration processes [5]. The NO_3_^−^ concentrations are below the MAC (50 mg/L) and vary between 4.00 and 24 mg/L (Table 1). Sources of NO_3_^−^ are intense agricultural practices, sewage and septic tank leakage, manure and contaminated sludge deposits and microbial decomposition. The geological and hydrogeological structure influences the groundwater contamination with NO_3_^−^ as well. Cancer and methemoglobinemia in infants are two diseases caused by the consumption of waters rich in NO_3_^−^ [46].

The PO_4_^3−^ concentrations are lower than 0.05 mg/L (G2 and G9), given the intense use of fertilizers and detergents. The presence of PO_4_^3−^ has a geogenic origin, as well. A high amount of phosphate lowers the quality standard of clean water by altering the colour and taste of the water [47]. The SO_4_^2−^ content is lower than 250 mg/L (MAC), ranging between 5.9 and 132 mg/L. Potential sources of this compound are represented by acid rain, volcanic eruptions, organic matter and mineral decomposition or burning, and the oxidation process of minerals and water–rock (gypsum and lignite) interactions. Water rich in SO_4_^2−^ could alter in taste and cause severe diarrhoea, but it does not present toxicity to consumers [48]. HCO_3_^−^ ranges between 109 and 737 mg/L. According to Rupias et al. [49], sources of HCO_3_^−^ are chemical reactions which involve silicate minerals and the presence of atmospheric CO_2_ and CO_3_^2−^ minerals.

Cl^−^ was below the MAC (250 mg/L). Sample G2 has the highest value (207 mg/L) and sample G3 has the lowest value (1.2 mg/L). The high chloride concentrations are widely dispersed in all types of rocks and are related to their natural occurrence in the aquifer’s geological strata [47]. Cl^−^ is characterized by mobility commonly distributed in all types of rocks from the geological strata of boreholes. The presence of Cl^−^ in drinking water is essential for electrolyte balance, while a high amount increases the potential chemical aggressiveness [50]. Water–rock (magmatic rock dissolution) and water–soil interaction, the use of intense chemical fertilizers, irrigation, wastewater infiltration, domestic sewage and waste deposit leakage are sources of high amounts of Cl^−^ [47,49]. Samples are characterized by F^−^ ranging between 0.1 and 0.2 mg/L, which is lower than the MAC (0.5 mg/L). Consuming waters rich in F^-^ could lead to serious health issues, such as enzymatic metabolism alteration or growth inhibition [48].

### 3.2. Trend of Metal Composition in the Studied Samples

The chemistry of studied water samples, based on the microelements and cations, is indicated in Table 2. The entire periodic system of all 118 elements was analysed, but only the elements with amounts above the detection limits were presented in this paper. The average values and standard deviation of the 12 measurements for each 10 sampling points were also exposed.

Na varied between 2.2 mg/L and 27.5 mg/L, with a mean value of 13.5 mg/L. K varied between 0.5 mg/L and 6.9 mg/L, with a mean value of 2.1 mg/L. The interaction between water and rocks (metamorphic and magmatic) and the alteration of minerals enriches the water with Na and K. Agricultural activities and the use of salt on roadways are sources of K and Na in the water [51]. If consumed, water with high amounts of K and Na causes digestive, heart and nervous diseases [51]. High amounts of K and Na are indicators of water pollution, having natural and anthropogenic sources represented by rock dissolution, erosion of salt deposits, industry practices and the use of salts for defrosting the roads [49,51,52].

Ca is below 100 mg/L. G7 is characterized by the highest amount of Ca, and the lowest concentration characterizes G9. Ca is essential in different biological processes, including bone development and cell wall permeability [10]. Mg varied between 1.0 mg/L and 10.8 mg/L, having a mean value of 3.72 mg/L. Sources of Ca and Mg are represented by natural processes, the interaction of water with the soil and rocks and dissolution processes [49]. Mg is also essential for positive functioning of the heart and is implicated in enzymatic systems, biological processes and haematopoiesis [53]. Ba was determined in water samples, ranging from 4.9 μg/L (G9) to 67.9 μg/L (G10), and the average was 25.5 μg/L. Potential sources are represented by the presence of volcanic, alkaline-igneous and granite rocks. A low pH influenced the high amounts of Ba in the water samples [25]. Ba has the ability to deposit in the bone and muscle systems, resembling the Ca amount [48]. The Sr amount ranged between 11.3 μg/L (G9) and 226 μg/L (G7, exceeding the MAC of 200 μg/L). The mean value is 51.8 μg/L, which could be attributed to the geogenic origin (Sr is a ubiquitous and natural element), soil and rock weathering process, depending on the infiltration conditions, sedimentation rate and rock typology [19,20,54]. Sr in water is toxic when the concentration surpasses the limit of 7000 mg/L due to its ability to substitute calcium in bones, potentially impacting bone growth and strength [19,20]. Sr is strongly corelated to Mg, Ca, HCO_3_^−^ and TDS [19].

**Figure 2:**
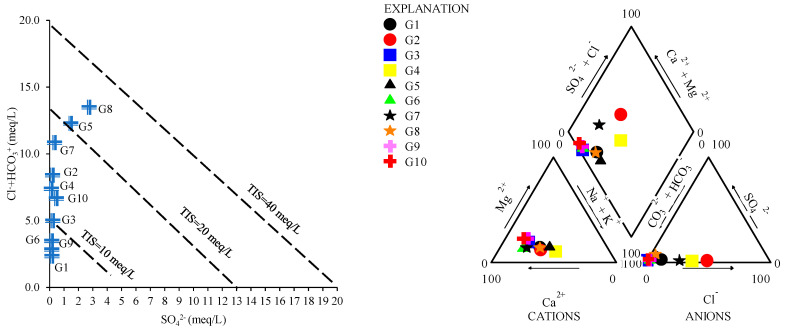
TIS (Total Ionic Salinity) and Piper diagrams of groundwater samples (G1–G10).

Given the salinity of water, the TIS (Total Ionic Salinity) diagram was used, based on the Cl^−^, HCO_3_^−^ and SO_4_^2−^ concentrations and the ratio between them (Figure 2). Generally, groundwater samples have a TIS lower than 40 meq/L. G1, G6 and G9 are characterized by a TIS lower than 10 meq/L, while G5 and G8 are considered to have the highest determined TIS (20 meq/L).

**Figure 3:**
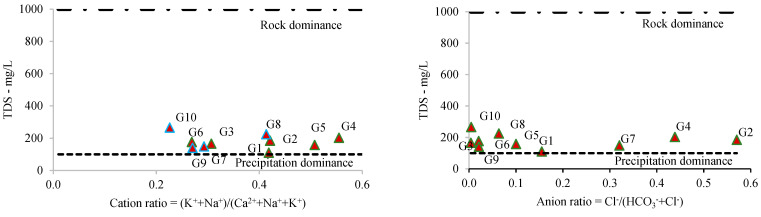
Gibbs diagrams of groundwater samples (G1–G10).

A Piper plot was built based on the major ions, which indicates the main typology of groundwater. According to the cation trilinear diagram, the majority of samples are grouped into the calcium typology, while G4 and G5 are sodium types (Figure 3).

The anion trilinear plot indicates that except for G2, which has no dominant typology, all samples are characterized as bicarbonate. Both trilinear plots are reflected in the diamond plot, which reflects the main water typology, indicating that except for G2 (mixed typology), all groundwater samples are classified as CaMgHCO_3_ waters.

Given the major cation amounts and the ratio between the TDS and cations and anions, the Gibbs plot was applied to establish the dominant source. The Gibbs diagrams indicated that the main ion sources are characterized by rock dominance (Figure 3). Although, the chemistry of G1, based on the plots, may have a precipitation origin.

The content of microelements is relatively high but does not exceed the MACs, related to drinking water regulation (Table 2). Industrial, mining and agricultural activities are the main sources of high amounts of toxic trace elements. Groundwaters have relatively significant amounts of Mn and Fe, probably due to natural geological sources (dissolution of amphibole, pyroxene and olivine) [49]. Mining activities enrich waters with Mn, which, after ingestion or dermal contact, can pose negative health effects [18]. The mean value of Fe is 29 µg/L and 19 µg/L for Mn, with the highest concentrations obtained in F10 and G7, but below the MAC. High amounts of Mn ingested through water can seriously affect the nervous system, affecting cognitive functions and motor ability [55]. A significant amount of Fe indicates the presence of organic colloids and humic materials. After implication in a redox reaction, Fe alters the properties of water, altering the odour and taste [49]. In high amounts, Fe could affect human health, causing Parkinson’s, Alzheimer’s disease and affecting respiratory, neurologic or cardiologic systems. On the other hand, proteins, enzymes and haemoglobin are dependent on small amounts of Fe [56]. Al ranged between 1.3 and 5.1 µg/L, with sources related to mining waste generally present in the mountain areas of Romania or acid rain dissolving minerals from soil and leaking to groundwater sources. If consumed, bioaccumulation of Al, known as a neurotoxin, in humans could affect the nervous system (sclerosis, Parkinson, Alzheimer), but also liver, brain, heart and bone systems, and could cause anaemia, asthma and gastrointestinal inflammation [50,57]. According to Weisner et al. [57] waters with amounts of Al of 0.1 mg/L day^−1^ and higher than 0.1 mg/L day^−1^, in the case of elder people, could increase the risk of cognitive impairment. 

As ranged between 1.3 and 5.1 µg/L, with a mean of 2.3 µg/L, indicating no contamination of water since the MAC (10 µg/L) was not exceeded. As is found naturally in water, in different shapes as oxides, sulphur and sodium and calcium salts. As is associated with granitic, volcanic and sedimentary rocks. Its amount and mobility depend on the geological characteristics of the soil and rocks [58]. If the geochemical conditions favour the dissolution of As in water, naturally occurring As and contamination will occur [59]. If contaminated water with this element is consumed, As is rapidly absorbed by the gastrointestinal system and starts to be metabolized, causing different and severe adverse effects in the short and long term, such as hepatic lesions, vomiting, diarrhoea, hypertension, cardiovascular diseases, diabetes, muscular weakening, cancer and death [13,36,58].

Cu is below the MAC (20 μg/L) by a factor of almost ten, with an average of 9.44 μg/L. Sources of Cu in water are related to its mobility from soil or rocks to water and acid rain, rock degradation and mining, depending on the organic matter, metallic oxides and hydroxides or clay minerals [60,61]. Cu is released into the water after chemical reactions occur due to the presence of Cu_2_S ores in the aquifer bedrock [60,61]. Cu does not bioaccumulate in human bodies, but it can be ingested in high amounts and become very toxic, causing headache and stomach pains, vomiting and diarrhoea, as well as irritation of the nose and eyes, kidney and liver lesions, coma and even death [61].

Cd is relatively high (in G7 = 2.3 µg/L) but below the MAC. Mining activities, burning emissions, the intense use of fertilizers and rocks rich in carbonates, sulphur or phosphates are general sources of Cd in water [16]. Water rich in Cd causes diarrhoea, complications at the skeleton level, kidney and renal disfunction, depression and affects muscular function [62].

Cr and Co were below 6.5 µg/L, with an average of 3.85 µg/L and 3.27 µg/L. Waters are enriched with Cr mainly from natural sources, with hydrogeological, geological and anthropogenic origins [14,56]. Gastric, intestinal and liver issues occur if water rich in Cr (highly toxic heavy metal) is used as drinking water [56]. Chronic exposure to Co could lead to pneumonia, fibrosis, oxidative stress, DNA damage and cancer [20,56,63]. On the other hand, at low concentrations, Co has significant importance in enzymatic and coenzymatic activities [20].

Zn was below 40 µg/L, with the highest amount detected in G3, compared to the majority of heavy metals, which had the highest amount in G7 and G10, perhaps due to a higher groundwater interaction with the bedrock. Sources of Zn, known for their high mobility, are represented by magmatic rocks, metal processes and coal combustion under the influence of the water’s pH, P availability, amount of organic matter and inorganic carbon [33,41]. Zn represents an essential mineral for a healthy life, although a significant amount is corelated to anaemia, vomiting or cramps, diabetes and renal and hepatic lesions of chronic diseases [56,64].

Pb is relatively elevated in G7, lower than 7.5 µg/L, and G5, lower than 5.5 µg/L, both lower than the MAC (10 µg/L) concerning drinking water quality. Pb naturally occurs through the mineralization and mobility of carbonate rocks through anthropogenic (mining, metal industry) sources in the groundwater [40,63,65]. If used as drinking water sources, waters rich in Pb can negatively and irreversibly affect the functioning of the kidneys, bones, thyroid and nervous system due to their bioaccumulate, toxicity and oxidative degradation abilities [33,49,66,67]. Considering the actual and largely spread issue (there are around 18 million people affected by poisoning) and considering the high levels of Pb in the drinking water sources, it is mandatory to determine, prevent and control the contamination with this toxin [67,68].

Ni ranges between 0.7 and 3.9 µg/L, with a mean of 2.01 µg/L, and is influenced by the pH, depth and soil composition. Ni is naturally found in human body tissues, as it is an essential constituent of blood, gene synthesis, endocrine system and enzyme activity. Nevertheless, if ingested from external sources (fossil fuel combustion, mining, municipal and industrial waste, dissolution of rich rocks in ore), pathological effects can occur due to its disruptor impact on insulin secretion and glucose metabolism [69,70,71]. According to the U.S. EPA, Ni is a significant contributor to water pollution, and therefore, it is mandatory to be monitored and for an associated health risk assessment to be implemented [71].

Se was below 6.5 µg/L, with an average of 3.43 µg/L. The dissolved matter, microbiological activities and redox potential influence the occurrence of Se in the waters, as well as anthropogenic activities [72]. A high amount of ingested Se through water consumption can cause cirrhosis, diabetes and cancer [72].

Sc is naturally found in groundwater. Sc ranged between 1.1 and 3.4 µg/L in the studied waters. The intense erosion chemically decomposes and releases Sc from the protolith ore, although the most favourable rock in forming Sc deposits is represented by ultramafic and felsic rocks. In water, a significant amount of Sc can influence and alter human health, causing fever, gastroenteritis and viral hepatitis [73]. The mean concentration of Rb was 2.69 µg/L. Leakage of sterile rocks enriches groundwaters with Rb. After water enrichment, Rb is deposited, as it is geochemically inert [41]. Health can be altered after long term exposure to Rb (locomotor ataxia, nervousness or weight loss) [21]. The high content of Li (G7, 53.4 µg/L) in the studied water samples indicated strong water/rock interactions. Li is the lightest alkali-metal naturally hosted in amphiboles, micas, granites and schists minerals [74]. If it exceeds the MAC, it affects human health, causing vomiting and renal and circular issues [52].

**Figure 4:**
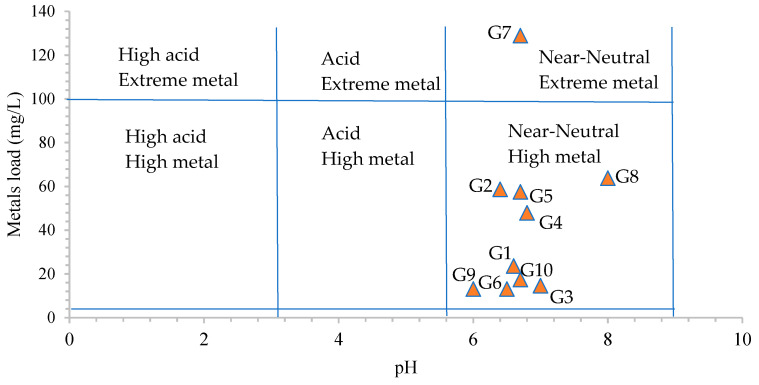
Ficklin–Caboi diagram.

Based on the pH and the metal load (all elements determined in this study—Table 2) in the samples, the Ficklin–Caboi plot was prepared (Figure 4). According to this plot, most of the samples were classified into the near-neutral high metal class, while only the G7 sample was classified into the near-neutral extreme metal class. This indicates that the studied metals present relatively high mobility in the aqueous phase.

### 3.3. Heavy Metal Pollution and Risk Assessment

**Figure 5:**
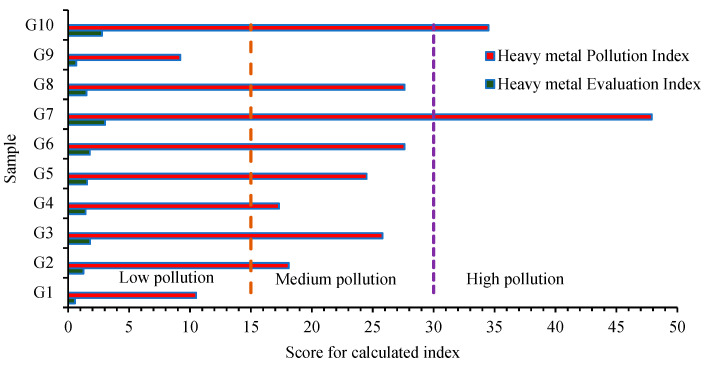
Heavy metal pollution evaluation for samples (G1–G10) according to the pollution indices scores.

Based on the relatively high amounts of some heavy metals (As, Cr, Cd, Cu, Fe, Mn, Ni, Pb and Zn), a pollution assessment was implemented to determine the degree of heavy metal pollution. The applied heavy metal indices indicated that the studied groundwater samples were characterized by three heavy metal pollution degrees (Figure 5).

According to the HPI and HEI scores, ranging between 9.2 and 47.9 and 0.56 and 3.02, the majority of samples (>50%) have a medium heavy metal pollution level, while samples G7 and G10 (20%) have a high pollution degree. The high scores are correlated to the high concentrations of Fe, As, Cd, Co, Mn and Pb. Other studies at the national level (central part of Romania) indicated that due to the former industrial activities, high amounts of heavy metals enrich the groundwater sources. According to the pollution index scores, the studied groundwater sources were classified into two pollution level categories. The results ranged between 2.0 and 226, indicating a low heavy metal pollution level and a high heavy metal pollution level. The pollution index was applied for the following heavy metals: Cd, Cr, Cu, Fe, Mn, Ni, Pb and Zn [75]. Studies from the northern part of Romania indicated that the studied groundwaters were classified into three different heavy metal pollution levels. Based on the pollution index scores, samples are characterized by low heavy metal pollution (heavy metal pollution index (HPI) and heavy metal pollution evaluation index (HEI) scores < 10.0), medium heavy metal pollution index (10 < HPI and HEI < 30), and also high heavy metal pollution (30 < HPI < 45). The pollution indices were calculated for As, Al, Cd, Cr, Cu, Fe, Mn, Ni and Zn, and the results ranged between 1.52 and 41.2 [76].

The highest intake was determined for Mn and Cu, with the following trend: Mn in G10, followed by G7 > G3 > G2 > G6, Cu in G5 > G6, Mn in G4, and Cu in G3 > G10 > G4. The lowest intake characterized Cd in G3 < G4 < G1 < G2 < G5 and As in G3 < G4 < G2 < G9 < G1. In other national studies, CDI ranged between 9.1 × 10^−6^ mg/kg/.day and 2.1 mg/kg/day in the north of Romania, 3 × 10^−4^ mg/kg/day and 7.2 mg/kg/day in the central part of Romania [75,76]. According to Moldovan et al. [77] in the eastern part of Romania, the CDI scores were lower than 1.28 µg/kg/day.

A non-carcinogenic risk assessment of As, Cd, Cu and Mn was determined through the water ingestion pathway. The results are indicated in Table 3. The highest HQ scores were obtained in the case of As, ranging between 0.124 and 0.314, followed by Cu (1.1 × 10^−3^–0.10) and Mn (3.5 × 10^−4^–9.9 × 10^−3^).

The HI (varying between 0.139 and 0.510) did not exceed the threshold limit of 1.0, which indicates that it is safe to use the studied groundwater samples as drinking water sources. Considering all the results obtained in the non-carcinogenic risk assessment, which were below the threshold limit, cancer risk was not evaluated for exposure to As, Cd, Cu and Mn. Correspondingly, the As and heavy metals determined in the water samples were below the MACs concerning the drinking water sources; therefore, we presume that all results will be below the threshold limit as well. 

In different studies from Romania, HQ was below 0.72 (groundwater collected from the central part of the country) and 0.08 (groundwater collected from the north), indicating no non-carcinogenic risks from heavy metals through water ingestion [75,76]. In the eastern part of the country, the HQ scores ranged between 2.3 × 10^−5^ and 4.3, mainly due to the heavy metals enriched by natural sources and geogenic processes [60].

## 4. Conclusions

The obtained results, considering the studied water samples from the Gutai Mountains, Romania, indicated that based on the metal load and pH, the majority of samples are classified as the near-neutral high metal class. It was observed that the waters were rich in NH_4_^+^, and in most of the samples, the MAC was exceeded two times, except for samples G3 and G8–G10. A potential source of the high amount of NH_4_^+^ could be anthropogenic activities, such as the presence of faecal deposits and the use of fertilizers based on nitrogen. Generally, groundwaters were characterized by relatively high salinity, based on the total ionic salinity diagram showing rock dominance, and only one sample presented precipitation dominance. According to the Piper diagram, based on the major ions’ concentrations, samples are classified as CaMgHCO_3_ water types. The presence of inorganic dissolved substances and loaded sewage sludge rich in heavy metals caused enrichment in NH_4_^+^, NO_3_^−^ and Sr and similarly high electrical conductivity in sample S2. Using those waters as drinking sources, the inhabitants are susceptible to negative effects on health, such as diarrhoea, hypertension or other cardiovascular affections. Sample G3 is rich in Cu and Zn, probably due to the content of organic matter, acid rain, metallic oxides and hydroxides. The sample with the highest As, Ba, Ca, K, Na, Mg and Sc concentrations is represented by G7. High amounts of Al, Rb and DO were determined in G9. The chemical composition of samples G7 and G9 is influenced and altered by the presence of mining waste, generally present in the mountain areas of Romania. This observation is related to the qualitative results obtained regarding the heavy metal pollution indices. The results indicated that more than 50% of samples are characterized by moderate heavy metal pollution level and 20% by high heavy metal pollution level. The non-carcinogenic risk assessment scores were lower than the threshold limit, which indicates that if the water is ingested, no non-carcinogenic risk from heavy metals can occur. 

Based on all of the data results obtained in this research, the population and the local competent authorities should be informed in order to adopt and implement risk management strategies. Further studies on toxicity aspects need to be conducted and implemented in order to explore the negative effects on human health after long-term exposure to heavy metals and to assess the potential human health risk related to nitrogen compounds, which were determined to be rich in the studied water samples.

## Figures and Tables

**Table 1:** Physico-chemical composition of water samples and MACs concerning the drinking water quality [25]. The results are expressed as mean ± standard deviation (n = 12 (monthly measurement in 2022)).

	Samples	G1	G2	G3	G4	G5	G6	G7	G8	G9	G10	Mean	MAC *
Parameter													
EC (μS/cm)		150 ± 15	663 ± 50	77 ± 8	454 ± 50	327 ± 30	332 ± 33	68 ± 7	82 ± 8	72 ± 7	125 ± 12	232	2500
DO (mg/L)		8.3 ± 0.8	8.2 ± 0.8	8.6 ± 0.9	8.0 ± 0.8	8.4 ± 0.8	8.3 ± 0.8	7.7 ± 0.8	8.5 ± 0.9	8.9 ± 0.8	6.9 ± 0.7	8.18	-
pH		6.6 ± 0.5	6.4 ± 0.5	7.0 ± 0.5	6.8 ± 0.5	6.7 ± 0.5	6.5 ± 0.5	6.7 ± 0.5	8.0 ± 0.5	6.5 ± 0.5	6.7 ± 0.5	6.79	6.5–9.5
ORP (mV)		208 ± 21	188 ± 20	196 ± 20	223 ± 22	212 ± 21	221 ± 22	179 ± 18	192 ± 20	234 ± 23	199 ± 20	205	-
T (NTU)		4.4 ± 0.4	4.8 ± 0.5	**5.2** ± 0.5	4.4 ± 0.4	**5.6** ± 0.5	**5.4** ± 0.5	4.3 ± 0.4	3.2 ± 0.3	2.5 ± 0.2	3.8 ± 0.4	4.4	<5
TDS (mg/L)		110 ± 11	185 ± 19	166 ± 17	203 ± 20	158 ± 16	177 ± 18	149 ± 15	225 ± 23	141 ± 14	266 ± 27	178	-
NH_4_^+^ (mg/L)		**0.82** ± 0.08	**0.84** ± 0.08	0.40 ± 0.04	**0.65** ± 0.07	**0.52** ± 0.05	**0.60** ± 0.06	**1.20** ± 0.01	0.28 ± 0.03	0.11 ± 0.01	0.17 ± 0.02	**0.56**	0.5
NO_3_^−^ (mg/L)		23.1 ± 2.3	23.7 ± 2.4	20.5 ± 2.0	21.3 ± 2.1	13.9 ± 1.4	14.6 ± 1.5	22.5 ± 2.3	18.1 ± 1.8	4.1 ± 0.4	4.0 ± 0.4	16.6	50
NO_2_^−^ (mg/L)		0.05 ± 0.01	0.04 ± 0.01	0.12 ± 0.01	0.21 ± 0.02	0.07 ± 0.01	0.44 ± 0.04	0.25 ± 0.03	0.28 ± 0.03	0.45 ± 0.05	0.38 ± 0.04	0.23	3
PO_4_^3−^ (mg/L)		0.33 ± 0.03	0.05 ± 0.01	0.12 ± 0.01	0.21 ± 0.02	0.09 ± 0.01	0.40 ± 0.05	0.15 ± 0.02	0.09 ± 0.01	0.05 ± 0.01	0.33 ± 0.03	0.18	0.2
SO_4_^2−^ (mg/L)		7.8 ± 0.8	9.5 ± 0.9	10.7 ± 1.1	5.8 ± 0.6	70.3 ± 7.0	6.9 ± 0.7	16.8 ± 1.7	132 ± 12	7.3 ± 0.7	22.6 ± 2.3	29.0	250
CO_3_^2−^ (mg/L)		85 ± 9	106 ± 11	186 ± 18	121 ± 12	455 ± 45	123 ± 12	223 ± 22	556 ± 60	97 ± 10	257 ± 25	221	-
HCO_3_^−^ (mg/L)		109 ± 10	156 ± 15	303 ± 30	192 ± 20	628 ± 63	205 ± 21	365 ± 36	737 ± 70	167 ± 17	403 ± 40	327	-
F^−^ (mg/L)		0.15 ± 0.02	0.13 ± 0.01	0.11 ± 0.01	0.17 ± 0.02	0.15 ± 0.02	0.10 ± 0.01	0.19 ± 0.02	0.16 ± 0.02	0.12 ± 0.01	0.10 ± 0.01	0.14	1.5
Cl^−^ (mg/L)		20 ± 2	207 ± 20	1.2 ± 0.1	150 ± 15	70 ± 7	4.3 ± 0.4	172 ± 17	50 ± 5	3.6 ± 0.4	1.9 ± 0.2	68	250
Br^−^ (mg/L)		0.7 ± 0.1	3.5 ± 0.3	0.8 ± 0.1	0.9 ± 0.1	1.3 ± 0.1	1.0 ± 0.1	65 ± 7	1.8 ± 0.2	2.4 ± 0.2	1.0 ± 0.1	7.8	-

MAC * = the maximum allowable concentration for drinking water stipulated by Law 311/2004 and Law M.O. No. 458/2002, Romania, in accordance with the Law on the quality of drinking water (552/29 July 2002).

**Table 2:** The annual median values of metals in water samples and guidelines according to 98/83/EC Council Directive. Data are expressed as a mean ± standard deviation (n = 12).

Samples Parameter	G1	G2	G3	G4	G5	G6	G7	G8	G9	G10	Mean	MAC
Ca (mg/L)	12.5 ± 1.2	31.5 ± 3.1	8.8 ± 0.9	19.9 ± 2.0	25.8 ± 2.6	8.7 ± 0.9	83.2 ± 8.3	34.2 ± 3.5	8.1 ± 0.8	11.3 ± 1.1	24.4	100
Mg (mg/L)	2.0 ± 0.2	4.1 ± 0.4	1.7 ± 2.0	3.0 ± 0.3	4.9 ± 0.5	1.0 ± 0.1	10.8 ± 1.1	5.3 ± 0.5	1.9 ± 0.2	2.5 ± 0.3	3.7	50
Na (mg/L)	7.9 ± 0.8	20.9 ± 2.0	2.4 ± 0.2	23.1 ± 2.3	24.1 ± 2.4	2.2 ± 0.2	27.5 ± 2.8	21.5 ± 2.2	2.5 ± 0.3	2.4 ± 0.2	13.5	200
K (mg/L)	1.1 ± 0.1	2.0 ± 0.2	1.5 ± 0.1	1.7 ± 0.2	2.5 ± 0.3	1.0 ± 0.1	6.9 ± 0.7	2.6 ± 0.3	0.5 ± 0.1	0.9 ± 0.1	2.1	10
Al (μg/L)	2.4 ± 0.2	1.7 ± 0.1	2.1 ± 0.2	1.5 ± 0.1	1.9 ± 0.2	1.3 ± 0.1	3.1 ± 0.3	3.6 ± 0.4	3.3 ± 0.3	5.1 ± 0.5	2.6	200
As (μg/L)	1.6 ± 0.2	1.5 ± 0.2	1.3 ± 0.1	1.4 ± 0.1	1.8 ± 0.2	3.3 ± 0.3	5.2 ± 0.5	3.1 ± 0.3	1.5 ± 0.1	2.3 ± 0.2	2.3	10
Ba (μg/L)	12.6 ± 1.3	36.1 ± 3.6	22.4 ± 2.2	16.2 ± 1.6	15.9 ± 1.6	11.6 ± 1.2	59.3 ± 6.0	7.8 ± 0.8	4.9 ± 0.5	67.9 ± 0.7	25.5	700
Cd (μg/L)	0.5 ± 0.1	0.8 ± 0.1	1.6 ± 0.2	0.4 ± 0.1	1.0 ± 0.1	1.8 ± 0.2	2.3 ± 0.2	1.6 ± 0.1	0.3 ± 0.1	1.5 ± 0.2	1.2	5
Co (μg/L)	1.1 ± 0.1	3.3 ± 0.3	1.6 ± 0.2	1.9 ± 0.2	2.9 ± 0.3	4.8 ± 0.5	5.9 ± 0.6	4.5 ± 0.5	1.5 ± 0.2	5.2 ± 0.5	3.3	-
Cr (μg/L)	3.4 ± 0.3	4.5 ± 0.5	2.5 ± 0.3	3.3 ± 0.3	6.2 ± 0.6	4.3 ± 0.4	3.3 ± 0.3	2.9 ± 0.3	4.6 ± 0.5	3.5 ± 0.4	3.9	50
Cu (μg/L)	1.8 ± 0.2	2.2 ± 0.2	14.8 ± 1.5	12.2 ± 1.2	19 ± 0.2	16.1 ± 1.6	9.9 ± 1.0	1.5 ± 0.2	2.6 ± 0.3	14.3 ± 1.4	9.4	100
Fe (μg/L)	2.1 ± 0.2	5.8 ± 0.6	45.5 ± 4.6	38.5 ± 3.9	18.5 ± 1.9	33.4 ± 3.3	55.6 ± 5.6	23.2 ± 2.3	8.9 ± 0.9	58.7 ± 5.9	29.0	200
Li (μg/L)	9.8 ± 1.0	18.8 ± 1.9	13.2 ± 1.3	15.5 ± 1.6	27.2 ± 2.7	9.3 ± 0.9	**53.2** ± 5.3	33.1 ± 3.3	5.4 ± 0.5	10.6 ± 1.1	19.6	50
Mn (μg/L)	1.7 ± 0.2	21.3 ± 2.1	22.3 ± 2.2	15.2 ± 1.5	9.1 ± 0.9	20.4 ± 2.0	35.3 ± 3.5	11.9 ± 1.2	5.5 ± 0.5	48.3 ± 5.0	19.1	50
Ni (μg/L)	0.8 ± 0.1	1.9 ± 0.2	2.4 ± 0.2	1.6 ± 0.2	0.9 ± 0.1	1.7 ± 0.2	3.3 ± 0.3	2.4 ± 0.2	1.2 ± 0.1	3.9 ± 0.4	2.0	20
Sc (μg/L)	3.4 ± 0.3	1.7 ± 0.2	2.7 ± 0.3	1.2 ± 0.1	1.9 ± 0.2	1.1 ± 0.1	1.3 ± 0.1	2.1 ± 0.2	1.4 ± 0.1	1.8 ± 0.2	1.9	-
Se (μg/L)	2.3 ± 0.2	0.8 ± 0.1	4.1 ± 0.4	3.3 ± 0.3	5.1 ± 0.5	1.9 ± 0.2	1.2 ± 0.1	5.6 ± 0.6	3.8 ± 0.4	6.2 ± 0.6	3.4	10
Sr (μg/L)	25.5 ± 2.6	72.1 ± 7.0	13.3 ± 1.3	37.2 ± 3.7	44.5 ± 4.5	14.6 ± 1.5	**226** ± 22	55.2 ± 5.5	11.3 ± 1.1	18.2 ± 1.8	51.8	200
Pb(μg/L)	1.3 ± 0.1	2.9 ± 0.3	3.5 ± 0.4	4.4 ± 0.4	5.5 ± 0.5	1.8 ± 0.2	7.2 ± 0.7	3.2 ± 0.3	1.2 ± 0.1	5.8 ± 0.5	3.7	10
Rb (μg/L)	1.4 ± 0.2	1.9 ± 0.2	2.5 ± 0.3	1.6 ± 0.2	2.4 ± 0.2	1.8 ± 0.2	3.6 ± 0.4	2.5 ± 0.3	4.4 ± 0.4	4.8 ± 0.5	4.3	-
Zn (μg/L)	1.8 ± 0.2	2.8 ± 0.3	37.5 ± 3.7	2.7 ± 0.3	1.2 ± 0.1	7.3 ± 0.7	8.9 ± 0.9	10.5 ± 0.1	4.6 ± 0.5	5.4 ± 0.5	8.3	5000

**Table 3:** Non-carcinogenic risk assessment of heavy metals (As, Cd, Cu and Mn) through water ingestion pathway.

CDI										
mg kg^−1^ /day	G1	G2	G3	G4	G5	G6	G7	G8	G9	G10
Cd	1.4 × 10^−5^	2.3 × 10^−5^	4.6 × 10^−5^	1.1 × 10^−5^	2.9 × 10^−5^	5.1 × 10^−5^	6.6 × 10^−5^	4.6 × 10^−5^	9.0 × 10^−5^	4.3 × 10^−5^
As	4.6 × 10^−5^	3.7 × 10^−5^	3.7 × 10^−5^	4.0 × 10^−5^	5.1 × 10^−5^	9.4 × 10^−5^	1.5 × 10^−4^	8.9 × 10^−5^	4.3 × 10^−5^	6.6 × 10^−5^
Mn	4.9 × 10^−5^	6.4 × 10^−4^	6.4 × 10^−4^	4.3 × 10^−4^	2.6 × 10^−4^	5.8 × 10^−4^	1.0 × 10^−3^	3.4 × 10^−4^	1.6 × 10^−4^	1.4 × 10^−3^
Cu	5.1 × 10^−5^	4.2 × 10^−4^	4.2 × 10^-−4^	3.5 × 10^−4^	5.4 × 10^−4^	4.6 × 10^−4^	2.8 × 10^−4^	4.3 × 10^−5^	7.4 × 10^−5^	4.1. × 10^−4^
HQ										
	G1	G2	G3	G4	G5	G6	G7	G8	G9	G10
Cd	2.0 × 10^−5^	6.5 × 10^−5^	6.5 × 10^−5^	1.6 × 10^−5^	4.1 × 10^−5^	7.3 × 10^−5^	9.4 × 10^−5^	6.5 × 10^−5^	1.2 × 10^−5^	6.1 × 10^−5^
As	0.152	0.124	0.124	0.133	0.171	0.314	0.495	0.295	0.143	0.219
Mn	3.5 × 10^−4^	4.6 × 10^−3^	4.6 × 10^−3^	3.1 × 10^−3^	1.9 × 10^−3^	4.2 × 10^−3^	7.2 × 10^−3^	2.4 × 10^−3^	1.1 × 10^−3^	9.9 × 10^−3^
Cu	1.3 × 10^−3^	10.6 × 10^−3^	10.6 × 10^−3^	8.7 × 10^−3^	1.4 × 10^−2^	1.1 × 10^−2^	7.1 × 10^−3^	1.1 × 10^−3^	1.9 × 10^−3^	0.10
HI										
	G1	G2	G3	G4	G5	G6	G7	G8	G9	G10
	0.154	0.149	0.139	0.145	0.187	0.330	0.510	0.299	0.146	0.239

## Data Availability

Data are available on request from the corresponding author.
